# Label-Free Electrochemical Biosensors: An Updated Perspective Focused on Genosensing, Multiplexing, and Commercial Potential

**DOI:** 10.3390/bios16020098

**Published:** 2026-02-04

**Authors:** Jefferson H. S. Carvalho, Marcus A. S. Catai, Lucas V. Bertolim, Rafaela C. Freitas, Jessica R. Camargo, Laís C. Brazaca, Bruno C. Janegitz

**Affiliations:** 1 Laboratory of Sensors, Nanomedicine and Nanostructured Materials (LSNano), Federal University of São Carlos, Araras 13600-970, SP, Brazil; 2 São Carlos Institute of Chemistry (IQSC), University of São Paulo (USP), São Carlos 13566-590, SP, Brazil; marcuscatai@usp.br (M.A.S.C.); lais.brazaca@usp.br (L.C.B.)

**Keywords:** label-free detection, point-of-care biosensor, DNA genosensor, multiplex assays, commercial label-free

## Abstract

The increasing impact of infectious, cardiovascular and neurodegenerative diseases has intensified the demand for early and decentralized diagnostics. Label-free electrochemical biosensors are promising candidates, offering high sensitivity, low reagent consumption and miniaturizable, low-cost architectures for point-of-care (PoC) testing. This review summarizes advances in immobilization strategies, recognition elements such as DNA, antibodies, aptamers, and molecularly imprinted polymers, as well as electrode platforms including glassy carbon, screen-printed, and 3D-printed systems, with an emphasis on DNA biosensors, multiplexed configurations, and applications to disease biomarkers. Beyond analytical performance, we critically examine the barriers that keep most devices at the proof-of-concept stage, including bioreceptor stability and immobilization, limited validation in real samples, reliance on conventional materials, challenges in scalable manufacturing, transport, and storage, and the absence of fully integrated PoC systems. Finally, we discuss significant advances in sensitivity, reproducibility, and application to real samples, but note that translation to real-world use and commercialization remains limited.

## 1. Introduction

In view of the increase in human life expectancy and the technological and scientific advances in healthcare, it is important to note the higher prevalence of infections, cardiovascular diseases, cancer, and neurodegenerative disorders, among others. This relationship between longevity and increased disease prevalence supports the notion that the pursuit of early diagnosis is becoming a predominant requirement for improved clinical outcomes [1,2]. Well-established methods such as enzyme-linked immunosorbent assays (ELISA), polymerase chain reaction (PCR), plate culture, and immunofluorescence remain the standard, but they are still expensive, require specialized infrastructure and personnel, and involve multiple sample preparation and incubation steps that are poorly compatible with rapid and decentralized diagnosis [3,4,5]. Among the alternatives discussed in the literature, electrochemical biosensors offer relevant advantages, as they allow for miniaturization, low reagent cons umption, rapid response, and the use of inexpensive and potentially disposable platforms based on different biorecognition elements such as antibodies, nucleic acids, enzymes, aptamers, membrane receptors, etc. [6,7].

An electrochemical biosensor is a device capable of detecting compounds based on the presence of a biorecognition component that specifically binds to the analyte of interest, a physicochemical transducer that recognizes the signals generated by the biorecognition event, and an electronic apparatus that processes and amplifies them into measurable signals [8,9]. Two important classes of biosensors can be highlighted, namely labeled and label-free devices. In summary, labeled devices incorporate a biorecognition element, reporter element, or target molecule that is typically tagged with a component such as enzymes, nanomaterials, or radioactive/fluorescent molecules. Upon interaction with the analyte, this labeling leads to a change in the monitored signal, enabling sensitive detection of the target [10,11]. Label-free devices directly exploit intrinsic properties of the analyte, such as mass, charge, size, refractive index, dielectric permittivity, or changes in the electrical properties of the electrode–biorecognition layer interface, avoiding labeling and enabling more direct, faster, and potentially cheaper readouts.

Table 1 presents a comparative overview of electrochemical biosensors developed using labeled and non-labeled strategies for specific biomarkers of different diseases. The comparison demonstrates that neither approach is inherently superior, as both strategies exhibit strong analytical performance, including high sensitivity, acceptable reproducibility, wide linear ranges, and results comparable to gold-standard methods, as reported in the original studies. However, differences in experimental design, electrode configuration, sample matrix, and validation protocols limit direct quantitative comparison between studies.

Although labeled strategies may increase costs due to the need for additional biological components, none of the reported studies include cost analyses, which is generally a problem in publications and will be further discussed throughout the review. Furthermore, labeled approaches can add experimental complexity and preparation time, potentially impacting scalability and point-of-care applications. Overall, both strategies are analytically attractive, and their performance may vary depending on the application, with sensitivity and specificity favoring one approach over the other in specific contexts. Therefore, the choice between strategies with and without markers should be guided by the application requirements and practical constraints, and not by a supposed methodological superiority.

Given the increasing development of label-free electrochemical biosensors and their importance, it is worth mentioning relevant reviews on the subject that seek to contribute to the advancement of this area, as is the objective of this review. In this regard, the work of Cancelliere et al. [10], stands out, as it compiles in detail different methodologies, bioreceptors, and applications of label-free devices, focusing on their use in the food, clinical, and environmental areas. However, the authors highlight the important need for a survey on the influence and growth of multiplex sensors, indicating this as an area with great potential for future research. Chieng et al. [12] discussed advances in label-free devices for real-time determination of small molecules. Although they did not explore in depth the different types of biorecognition elements or the important studies on DNA biosensors, the authors addressed the use of different transducers capable of determining in real time molecules below 1 kDa. Al-Younis et al. [13] produced a review detailing the use of label-free devices with a focus on different materials integrated into field-effect transistors (FETs) for point-of-care (PoC) biomedical sensing probes, but without extending their discussion to sensors with simple and relatively low-cost fabrication.

Thus, it is clear that label-free electrochemical biosensors occupy a strategic space between traditional laboratory methods and portable devices, but require further studies of new trends and the pursuit of making them usable in everyday clinical diagnoses. They combine high miniaturization potential, compatibility with lab-on-a-chip architectures, and the possibility of real-time monitoring, but still face challenges related to robust biofunctionalization, fouling, standardization, and translation to real-world settings. This balance between advantages and limitations justifies the subsequent focus on specific architectures such as DNA genosensors and biosensors for emerging diseases, on multiplexing strategies, and on commercialization routes capable of transforming academic prototypes into widely accessible diagnostic tools.

Accordingly, this review article aims to discuss different topics related to the fabrication, application, and commercialization of label-free electrochemical biosensors, mainly impedimetric ones, highlighting recent works over the last 15 years on DNA genosensors, emerging diseases, and multiplexing, to provide a simple yet detailed overview of each of these aspects.

**Table 1:** Comparison between label-free and labeled biosensors for the same biomarkers of different diseases.

Disease Target	Strategy Type	Sensitivity	Specificity Compared to:	Reproducibility (RSD)	Linear Range	LOD	Ref.
Alpha-fetoprotein (cancer)	Label-free	High sensitivity, but without showing the calculated value.	Prostate-specific antigen, carcinoembryonic antigen, ascorbic acid	<6% (*n* = 4)	0.01 to 500 ng mL^−1^	0.007 ng mL^−1^	[14]
	Labeled		Human serum albumin, BSA, human chorionic gonadotropin, hepatitis B surface antigen, and carcinoembryonic antigen	3.68% (*n* = 6)	0.005 to 100 ng mL^−1^	0.0022 ng mL^−1^	[15]
Dengue non-structural protein 1 (NS1 DENV)	Label-free	0.044 μA∙mL∙ng^−1^	Fibrinogen, BSA, human serum albumin	5.1% (*n* = 3)	10 to 100 ng mL^−1^	8.23 ng mL^−1^	[16]
	Labeled	High sensitivity, but relating to the value obtained by the calculated LOD.	Norovirus-like particles, inactivated Influenza virus A/H1N1 and A/H3N2	10.2% (*n* = not informed)	0.001 to 1.0 ng mL^−1^	30 fg mL^−1^	[17]
SARS-CoV-2 (COVID-19)	Label-free	High sensitivity: the authors indicate this property by the behavior of the rate of decrease in the current increasing with increasing protein concentrations.	Human IgG, SARS spike glycoprotein, COVID-19 nucleocapsid protein	3.88% (*n* = 5)	0.001 to 10 ng mL^−1^	0.003 ng mL^−1^	[18]
	Labeled	High sensitivity attributed to the excellent signal amplification effect of the secondary probes.	Mutant SARS-CoV-2 spike protein, non-target coronavirus spike proteins, BSA.	10% (*n* = 3)	0.0001 to 1000 ng mL^−1^	0.0005 ng mL^−1^	[19]
Superoxide dismutase 1 (neurodegenerative diseases)	Label-free	highlights the high sensitivity to the presence of added palladium nanoparticles as an alternative to AuNPs.	Glucose and saturated salts	8.03% (*n* = 6)	1.0 to 100 ng mL^−1^	0.72 ng mL^−1^	[20]
	Labeled	High sensitivity considering the LOD value.	Horseradish peroxidase, alkaline phosphatase, alanine, arginine, glutathione, others.	2.58% (*n* = 11)	0.1 to 1.0 μg mL^−1^	0.03 μg mL^−1^	[21]
Phosphorylated tau 181 (p-tau181) (Alzheimer’s disease)	Label-free	High sensitivity based on estimated plasma p-tau181 levels analyzed in patients.	Human amyloid beta 1–40, human amyloid beta 1–42	Not informed	0.001 to 1000 pg mL^−1^	0.92 fg mL^−1^	[22]
	Labeled	High sensitivity demonstrated by showing a lower LOD than the ELISA assay.	β-amyloid monomers, β-amyloid oligomers, BSA, human serum albumin	1.4% (*n*= not informed)	6.97 fg mL^−1^ to 100 ng mL^−1^	1.91 fg mL^−1^	[23]
Cardiac troponin I	Label-free	High sensitivity considering the LOD value.	Epidermal growth factor receptor, epithelial cell adhesion molecule, human epidermal growth factor receptor 2, programmed death ligand 1	0.24% (*n* = 5)	0.001 to 10 ng mL^−1^	0.00008 ng mL^−1^	[24]
	Labeled	High sensitivity related to the generation of active sites for antibody immobilization 2	Insulin, carcinoembryonic antigen, prostate-specific antigen and squamous cell carcinoma antigen.	2.22% (*n* = 5)	0.0005 to 10 ng mL^−1^	0.00017 ng mL^−1^	[25]

## 2. Origin and Early Development of Label-Free Electrochemical Biosensors

In the context of electrochemical biosensing, the term “label-free” was initially introduced in the sense of “direct electrochemical detection. One of the pioneering studies was reported by Green et al. [26] in 1984, who described a pyrolytic graphite electrode modified with human IgG for detecting the oxidation of superoxide dismutase (SOD) in aqueous solution at neutral pH. The concept of “label-free devices” was further established by Wang et al. [27] in 1999, who presented a label-free biosensor based on polypyrrole (PPy) films for in situ detection of DNA hybridization. In that work, oligonucleotide probes were shown to act as counter-anions during PPy film growth, enabling real-time monitoring of DNA hybridization reactions.

It is important to note that this is an expected pattern for electrochemical and non-electrochemical sensors. In this context, four detection mechanisms can be implemented for label-free electrochemical sensors, as detailed in the work of Rahn et al. [9]. The options may include blocking the electrode surface, causing a conformational change in the opening for the biorecognition unit to allow for greater access to the electrode surface, or altering the intercalation or electrostatic affinity of a redox probe for the biorecognition unit.

**Figure 1:**
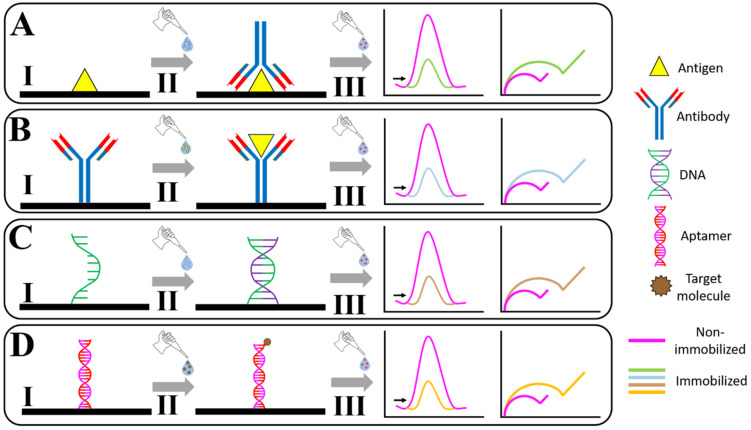
Schematic representation of the surface blocking and immobilization strategies. (**A**) Antigen immobilization: I—antigen immobilization on the electrode; II—antibody binding to form the immunocomplex; III—electrochemical measurement with a redox probe: higher charge transfer resistance/lower current (immobilized), lower resistance/higher current (non-immobilized). (**B**) Antibody immobilization: I—antibody immobilization; II—antigen binding; III—electrochemical measurement as described. (**C**) Label-free DNA immunosensor: I—immobilization of a specific DNA sequence; II—hybridization with the complementary strand; III—electrochemical measurement as described. (**D**) Label-free aptamer immunosensor: I—aptamer immobilization; II—ligand binding; III—electrochemical measurement as described. In all cases, redox probes indicate surface changes.

Considering the blocking mechanism for this review, this strategy typically involves a complex formed between a biorecognition element (such as antibodies, antigens, aptamers, DNA, etc.) and the analyte of interest. This complex hinders a redox probe’s access to the electrode, resulting in a substantial increase in the resistance to charge transfer, which enables the correlation between this signal and the analyte concentration [10]. Figure 1 presents a schematic illustration of the strategy for blocking access to the electrode surface and the immobilization process of antigens/antibodies, DNA, and aptamers.

However, with scientific advancements and, consequently, optimizations in the production of biosensors and new materials and components available for use, it is worth highlighting possible subdivisions of interest for label-free devices.

### 2.1. Bioreceptor Immobilization Strategies for Label-Free Devices

The success of label-free devices strongly depends on the selection of appropriate bioreceptor immobilization strategies, which in turn depend on the functionality and nature of the biorecognition element, as well as on the properties of the target analyte [28]. The main approaches include physical adsorption [29], chemisorption [30], self-assembled monolayers (SAMs) [31,32] and DNA-directed immobilization (DDI) [33].

In physical adsorption, bioreceptors are directly attached to the sensor surface predominantly through hydrophobic and electrostatic interactions. This approach is fast, inexpensive, non-destructive, and straightforward [10,28,34]. Chemisorption involves the chemical bonding of the biorecognition element to the substrate, providing a highly efficient and stable immobilization process [10,28]. Crosslinking agents such as NHS (N-hydroxysuccinimide) and EDC (1-ethyl-3-(3-dimethylaminopropyl)carbodiimide), or glutaraldehyde (GA) and cysteamine (Cys), are commonly used to establish strong linkages between components [10,28].

A more recent approach involves the use of polyA (poly-adenine), in which the bioreceptor is attached to a polyA tail for direct adsorption onto surfaces, particularly gold electrodes (GE), via electrostatic interactions [35,36]. This method is also effective on other electrode surfaces when supported by metallic nanoparticles such as gold (AuNPs) [37,38] and silver (AgNPs) [39,40]. SAMs represent another widely employed method, where thin films spontaneously form on specific substrates through chemisorption of short-chain organic alkanethiols. These films contain a “head” group that binds to the substrate, whereas the alkyl chain can be chemically functionalized [10,28]. Finally, DNA-directed immobilization involves conjugating antibodies to DNA oligonucleotide strands, which are subsequently hybridized onto the sensor surface [41].

Given the wide range of immobilization strategies, some that are commonly used should be highlighted because they demonstrate advantages such as relatively low cost, stability, reproducibility, and methodological simplicity. Immobilization in conjunction with nanomaterials and the electrode surface can provide interesting interactions that enhance the efficiency of label-free biosensors. The discussion of the main immobilization strategies, considering electrochemical sensors and recently published works, is a way to provide the field of label-free biosensors with new perspectives on the use of materials science and biological materials. It is of utmost importance that the joint advancement of both areas occurs to leverage the potential application of these types of devices to real problems involving pre-conceptual diagnoses and areas of need. Therefore, electrochemical biosensors have revolutionized the field of disease diagnostics and molecular research by offering highly sensitive, rapid, and cost-effective solutions for biomolecule detection. While DNA and protein-based receptors dominate the field, other alternatives emerge for bioreceptors, such as aptamers, peptides, and whole cells, open up new opportunities to broaden the potential of label-free devices. These approaches not only enhance the specificity and stability of biosensors but also diversify their applicability across emerging fields, including cancer biomarker detection, pathogen monitoring, and environmental analysis. Furthermore, before initiating a discussion focused on the types of label-free electrochemical devices and the importance of advancing research on the already widely studied genosensors, we briefly compiled a list of sensors with different bioreceptors that are on the rise.

In this sense, an antibody-based label-free device was constructed using EIS to detect calreticulin, a breast cancer biomarker in human serum. The biosensor featured a single-step electrodeposition of single-walled carbon nanotubes (SWCNTs) incorporating polymerization of an oxiran-2-yl methyl 3-(1H-pyrrol-1-yl) propanoate monomer (SWCNTs-PPepx nanocomposite) on a disposable indium tin oxide (ITO) electrode. It demonstrated high sensitivity and reliable clinical application for calreticulin quantification. The system showed satisfactory repeatability and reproducibility, making it a promising tool for rapid and accurate calreticulin analysis. As a result, they are increasingly recognized as reliable tools for applications ranging from disease diagnostics to environmental monitoring, offering a compelling balance between cost efficiency and analytical precision.

Aptamer-based sensors, in particular, can selectively bind to specific molecules, such as proteins or small metabolites, allowing for the development of label-free analytical methods. Aptamers are synthetic oligonucleotides that bind to target molecules with high precision, and their resistance to denaturation makes them a superior choice for many applications, especially in harsh conditions where traditional bioreceptors might fail [42]. An example is a multiplexed electrochemical aptasensor for cyanotoxins, as reported by Rhouati and Zourob [43]. Using a platform with AuNPs-modified carbon electrodes, this sensor enables the simultaneous detection of five cyanotoxins, including microcystin-LR, cylindrospermopsin, anatoxin-α, saxitoxin, and okadaic acid. The aptasensor detects the target toxins by monitoring conformational changes in specific aptamers, leading to changes in electron transfer, which can be measured electrochemically [43]. This innovation represents a significant step forward in label-free electrochemical biosensors, providing a robust and scalable solution for multiplex detection with potential applications in environmental monitoring, healthcare diagnostics, and food safety.

Another example is the work performed by Eteya et al. [44], which presents a novel, label-free method for detecting atrazine in solution using an aptamer-based sensor on a GCE. The WE was modified with chitosan and multiwalled carbon nanotube-graphene oxide nanocomposite (MWCNTs-rGO), facilitating aptamer attachment due to its amino and carboxyl groups. These sensors are particularly promising in disease diagnostics, where the accurate and rapid detection of emerging viruses and disease-associated proteins is critical. In the context of pandemics and emerging infectious diseases, such as COVID-19, these technologies have gained heightened relevance, enabling faster, more precise diagnostics [45,46,47].

Wang et al. [48] developed an electrochemical immunosensor for detecting tiamulin using a murine monoclonal antibody along with silver-graphene oxide (Ag-GO) and AuNPs nanocomposites. The synergistic combination of Ag-GO and AuNPs significantly enhanced the sensor’s conductivity, offering promising potential for developing advanced biosensor architectures. This design demonstrates the potential for monitoring veterinary antibiotics in animal-derived food products. Sethi et al. [49], which introduces a label-free biosensor designed to detect the Aβ1–42 biomarkers in plasma relevant to AD diagnosis. The sensor uses a dual-layer graphene and electrochemically rGO modified with 1-pyrenebutyric acid N-hydroxysuccinimide ester to enable H31L21 antibody attachment. Validation in spiked human and mouse plasma samples showed consistent results from brain analyses.

MIPs are synthetic materials engineered to mimic natural antibodies by creating specific binding sites within a polymer matrix. Producing MIPs involves forming monomer-template complexes, polymerization, and subsequent template removal, leaving molecular cavities that can selectively rebind with target molecules [50]. MIPs offer high chemical and physical stability, low cost, reusability, and excellent selectivity, making them ideal for applications in biosensing. Electrochemical sensors often use these materials to detect biomarkers such as proteins, nucleic acids, and small molecules. However, challenges like reduced mass transfer, non-specific binding, and difficulty fabricating consistent MIP layers hinder their widespread clinical application [50].

Rebelo and collaborators [51] described the development of a disposable electrochemical biosensor based on MIP to detect the enzyme α-amylase, a stress biomarker found in saliva. The sensor was designed for PoC applications, being portable and low-cost. The MIP was prepared using electropolymerization of pyrrole on a GE, with α-amylase acting as the template molecule. After enzyme removal, the MIP retains specific sites that recognize α-amylase in saliva samples. Moreover, the sensor is reusable and can be applied in clinical analyses.

However, despite their enormous potential, the commercialization of label-free electrochemical biosensors faces significant challenges [12]. The mass production of these devices requires standardized manufacturing processes to ensure consistency and reliability [52]. Additionally, rigorous validation in real-world conditions is essential to prove their effectiveness across diverse environments and applications. Until these issues are addressed, the widespread industrial-scale development of biosensors will remain limited [53]. For further discussion regarding the commercialization potential of label-free electrochemical devices, check Section 6 of this review.

In this sense, the evolution of electrochemical biosensors with other bioreceptors besides DNA holds immense promise, particularly in medical diagnostics and environmental monitoring. Overcoming the technical and industrial barriers will be key to unlocking their full potential and integrating these devices into mainstream healthcare and industrial processes. Table 2 summarizes various types of bioreceptors used for detecting specific targets, with each entry detailing the target, detection range, and LOD, highlighting the diversity in detection capabilities across different bioreceptors. In addition, exploring new designs and architectures may support the determination of different biomarkers for various diseases in the same label-free device while simultaneously seeking alternatives to facilitate the commercial application of these new sensors.

### 2.2. Electrochemical Devices in Label-Free Biosensors

The choice of immobilization route directly impacts the structure and homogeneity of the interfacial layer sensed by electrochemical techniques such as cyclic voltammetry (CV), differential pulse voltammetry (DPV), square wave voltammetry (SWV), and, most importantly for label-free formats, electrochemical impedance spectroscopy (EIS) [8]. In label-free systems, the interaction between the bioreceptor and the analyte often induces changes in the intrinsic properties of the analyte or modifies the electrode surface and its electrical characteristics. These changes are detected by a transducer element, which converts biochemical events into measurable signals. The signals are then processed and amplified by dedicated electronic components [9].

For that, electrochemical biosensors typically consist of a working electrode (WE) modified with a bioreceptor element responsible for binding to the target analyte and facilitating its detection. Common bioreceptors include antibodies, nucleic acids, aptamers, DNA enzymes, and protein scaffolds [67,68]. The most used WEs in label-free devices include glassy carbon electrodes (GCE) [69,70], boron-doped diamond electrodes (BDD) [71,72], GE [73,74], platinum electrode (Pt electrode) [75,76], carbon paste electrode (CPE) [77,78], screen-printed electrode (SPE) [79,80] and 3D-printed electrode [81,82].

The detection occurs through biochemical recognition of the formation of the immunocomplex, generating measurable signals, such as current, impedance, or potential. Due to the label-free approach, measurable electrical signals are produced directly by detecting changes in the electrical characteristics of the transducer surface caused by the formation of the specific immunocomplex [83]. Its advantages also contribute to the growth of its use and advances today. Among them, selectivity, sensitivity, and variety in the use of redox mediators and nanomaterials, among others, can be highlighted [84]. At this point, considering the infinity of devices manufactured for the most varied application areas, when focusing on clinical analysis, the sensitivity of electrochemical techniques stands out. Concomitantly, the selectivity offered by label-free methods supports the development and variety of combinations for this area of biosensing.

Therefore, different transducers can yield interesting analytical results across a wide range of targets. Zhang et al. [85] modified a GCE with an AuNP/coiled carbon nanotube/chitosan nanocomposite to detect carcinoembryonic antigen, achieving high selectivity and good performance in real samples. Recent studies include Chaturvedi et al. [86], who used an rGO/AuNP/polydopamine nanocomposite to determine Zika and Dengue NS1 for early detection, and Adu et al. [87], who applied a cobalt-hydrocarbon iron oxide nanocomposite (flame synthesis) to detect anti-A2 antibodies for visceral leishmaniasis, showing promising reproducibility and diagnostic potential.

Although GCEs have advantages such as high sensitivity, the potential for modification with a wide range of materials, and a significant amount of published work on label-free biosensors, some points should be highlighted. Among them, considering the possibility of a portable, miniaturized device with the potential for analysis in situations of need, this type of electrode has limitations that are relevant in the context of rapid clinical diagnoses and PoC, given the post-measurement surface poisoning, the need for prior treatment, and the instability of the immobilizer’s adhesion to the surface. In this sense, even considering all their analytical value, other tools are emerging to overcome some limitations. In this review, we do not intend to label conventional electrodes, such as GCE or BDD, as outdated or non-contributory to scientific advancement in label-free biosensors, but we bring the discussion of their limitations in a panorama of mass production and distribution for PoC analyses and in locations of need.

Also, SPEs or 3D-printed electrodes are devices with the potential to advance research on label-free biosensors for disease diagnosis. They are easy to produce in the laboratory, have relatively low production costs, are reproducible, can be miniaturized, and, consequently, are promising PoC devices. Table 3 presents the studies described below, the materials used, the targets, the electrochemical techniques employed, the linear range, and the limit of detection (LOD) obtained. We consider it important to highlight whether the analyses were performed on real or synthetic samples, as well as the potential portability of the system.

Considering label-free SPEs, a screen-printed carbon electrode modified with MWCNTs and electrodeposited AuNPs to detect α-synuclein was proposed, a key marker related to Parkinson’s disease [88]. Also, the polyethyleneimine and poly(4-sodium styrenesulfonate) were used to enable direct EIS determination of *Staphylococcus aureus*, associated with hospital-acquired infections [89]. Another example is the CO_2_ Laser-Fabricated immunosensor on polyimide sheets for serological detection, which demonstrated promising performance for dengue serum compared to a commercial strip test [90].

Metallic electrodes are frequently used in label-free immunosensors because they typically offer higher sensitivity and well-established selectivity, although at a higher cost. In this context, a label-free impedimetric gold sensor for Surfactant Protein B in amniotic fluid, functionalized with Sulfo-LC-SPDP, achieves good specificity and selectivity [91]. Also, a gold-electrode immunosensor was applied for osteoprotegerin, using a Ti_3_C_2_T_x_ MXene/carbon nanofiber composite with polyaniline nanoparticles [92]. As an alternative, 3D-printed electrodes are gaining attention due to low cost and accessible fabrication. For example, [93] the immunosensor using PLA/graphene filament was prepared to determine PARK7/DJ-1 for Parkinson’s diagnosis. Another example is the immunosensor with covalently immobilized antibodies on carbon black/PLA conductive filaments to detect the SARS-CoV-2 spike (S1) protein [94]. A skyscraper-shaped 3D-printed sensor with commercial carbon black/PLA filament was applied for detecting TNFα in feces, modified with thiophene-2-carboxylic acid after AuNP electrodeposition, highlighting 3D printing as a simple, robust route for diagnostic devices [95].

With this small sample of recently published works covering a wide range of diseases, some considerations can be raised to observe the current stage the label-free area is at and its possible direction. The first is the increased production of SPEs and their various modifications, resulting from the miniaturization of the system and the ease of in situ electrochemical measurements facilitated by the advancement of portable potentiostats. In the comparison in the table, we added a portability column indicating whether or not the authors presented this characteristic for the developed device. However, in addition to this, we established that for a device to be considered portable, it cannot only be capable of being moved from one location to another. The way the device would reach the places of need still requires in-depth studies; however, here we discuss the capacity of the device as a whole, that is, the sensor, the data collection equipment, and the way the results are obtained, being able to occur quickly and without the need for equipment with numerous components. Thus, a portable sensor is one that, with the aid of portable potentiostats, is miniaturized, easy to handle, and has the potential for post-measurement disposal.

Another advance to be pointed out is the analysis of real samples. Because these samples are difficult to access, as many laboratories do not have sufficient storage facilities, testing devices on real samples, with collection from patients affected by diseases or controls, becomes very difficult. One solution is to obtain commercial biological samples with the subsequent addition of the biomarker or the preparation of synthetic biological samples. In this context, it is possible to observe that recent studies demonstrate progress in this regard, whether using synthetic samples produced in the laboratory, commercial tests and real samples, or, in fact, partnerships with hospitals and collecting samples from patients. However, in the latter case, sample collection and measurement still occur in the laboratory and not through measurements performed directly at the collection site.

In general, it is possible to observe the advancement of label-free electrochemical biosensors in the use of smaller, simpler systems with a relatively lower cost, aiming at system portability and complementing the performance of tests that seek to approximate analyses on real samples to guarantee a future application in the clinical market.

## 3. Label-Free Genosensor

Although there is a wide range of biological recognition elements for label-free biosensors, those that use deoxyribonucleic acids (DNA) stand out because they play a vital role in carrying genetic information and regulating important physiological processes [96]. When immobilized on the transducer surfaces, these molecules enable the specific recognition of gene sequences through DNA probes [97]. This immobilization can be achieved through different pathways, involving either physical or chemical mechanisms. Chemically, single-stranded DNA (ssDNA) is immobilized onto the transducer surface through covalent bonding or specific chemisorption, which occurs via interactions between functional groups of the DNA and previously functionalized surfaces. Alternatively, physical immobilization of ssDNA can take place through adsorption, electrostatic interactions, or entrapment within polymeric matrices, relying on noncovalent forces between the DNA and the selected sensor material. In both approaches, hybridization between the immobilized probe and the complementary DNA target is enabled, leading to the formation of double-stranded DNA (dsDNA), which is subsequently converted by the transducer into a measurable signal [98]. Detection is typically achieved through hybridization, where a single-stranded DNA (ssDNA) probe binds to its complementary target sequence, forming a double-stranded hybrid (dsDNA) [97]. This hybridization-based detection ensures the development of stable, selective electrochemical biosensors that can be applied to diagnose various diseases, such as cancer, influenza, and coronavirus [97]. These diseases often involve genetic mutations or strain variations, which DNA-based biosensors can efficiently detect, providing a significant advantage over other biorecognition elements. Furthermore, these biosensors offer broad detection capabilities, extended functional lifespans, and cost-effective production [99]. Consequently, nucleic acids are pivotal in analytical device development, particularly in the biorecognition process through DNA/RNA fragments hybridization [100,101].

In this context, it is important to highlight that genosensors exhibit remarkable specificity and can discriminate oligonucleotide sequences with a single nucleotide substitution while detecting low nucleic acid concentrations [101]. This makes them widely applicable across biological and clinical fields. For example, Brazaca et al. [102], developed a genosensor by modifying a GE with a DNA strand containing the sickle cell disease mutation. The single base mismatch (GTG–Mutated; GAG-Healthy) was promptly detected by the label-free device using EIS. Additional advantages include their thermal and temporal stability, stable chemical structure, and ease of functionalization. These properties facilitate effective interaction with electrode surfaces and ensure proper assembly through various immobilization methods.

**Figure 2:**
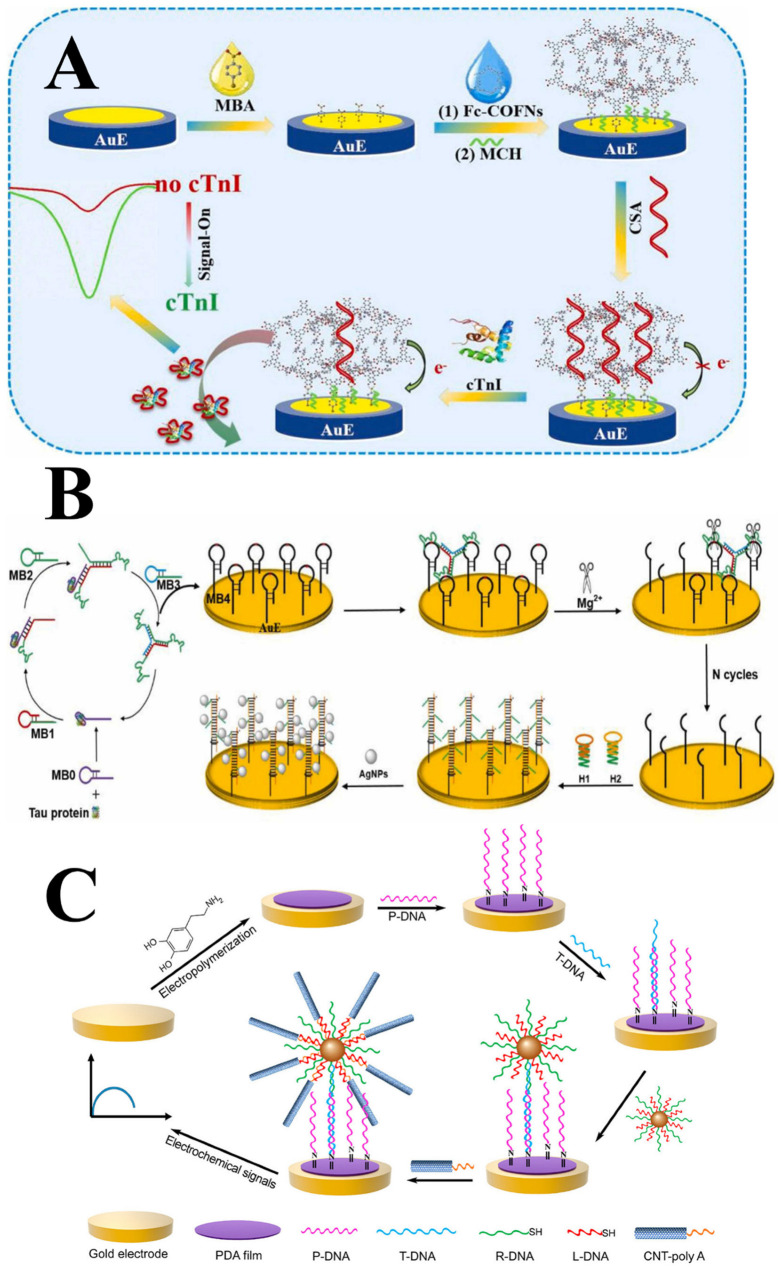
(**A**) Scheme for the Fc-COFNs as an electrochemical sensing platform for label-free analysis of cTnI. Reproduced with permission from Elsevier ref. [115]; (**B**) Scheme for the label-free electrochemical aptasensor principle based on the MNAzyme-driven tripedal DNA walker, triggered a hybridization chain reaction strategy for tau protein detection. Reproduced with permission from Elsevier ref. [116]; (**C**) Scheme of an Electrochemical DNA Biosensor Fabrication and Detection Process. Reprinted (adapted) with permission from ref. [117]. Copyright 2024 American Chemical Society.

Label-free genosensors can be manufactured by modification of the electrode surface with various materials, such as electroactive redox mediators [103,104], mimetic enzymes [105,106], nanomaterials [107,108,109], metallic nanoparticles [110,111], and carbon nanomaterials [112,113]. Aromatic redox mediators, such as methylene blue, have been widely studied. Methylene blue, when intercalated with G-triplex DNA (a type of non-canonical nucleic acid), has been used as a signal generator in microRNA detection through cylindrical carbon fiber microelectrodes, enabling the creation of label-free electrochemical devices [114]. Metal complexes can also be applied to these platforms as electroactive indicators. Ferrocene is one notable example, as used by Song et al. [115] (Figure 2A), who developed electroactive ferrocene-based covalent organic frameworks and aptasensors on a GE to detect cardiac troponin I (cTnI).

DNAzyme biosensors also allow for sensitive and selective detection of target molecules. Wei et al. [116] used a DNAzyme-driven tripedal DNA walker for the development of a label-free electrochemical sensor to detect the protein tau associated with Alzheimer’s disease (Figure 2B). These devices can also be fabricated using techniques such as the tripedal DNA walker drive, which Gong et al. [118] chose for the electrochemical biosensing of α-synuclein based on the Mg^2+^ signal. The highlight of these DNAzyme biosensors results in more stable platforms, resistance to mechanical and temperature changes, and no need for extensive manufacturing processes.

With the advancement of nanotechnology, the incorporation of nanomaterials into biosensing systems has grown due to their physical and chemical properties. Acting as modifiers that generate or amplify the signal in the determination of the target analyte and presenting the ability to functionalize their structures, nanomaterials allow the production of sensitive analytical devices while maintaining selectivity. Among them, metallic nanoparticles have been widely used to manufacture DNA biosensors, with AuNPs standing out. A label-free electrochemical DNA biosensor using gold nanocubes stabilized on a graphite screen-printed electrode (SPE) was proposed to detect a prostate cancer gene sequence [43]. Other DNA platforms combine nanomaterials, such as ZnO/AuNPs on a GCE for thiolated *Mycobacterium tuberculosis* DNA, in general, metallic nanoparticles improve sensitivity/selectivity and add biocompatibility [119].

The production of hybrid nanocomposites may be an alternative for achieving more sensitive biosensors. Based on this, it is possible to find recent works in which nanoclusters of AuNPs and CNTs were used to produce a DNA biosensor (Figure 2C) [117]. The authors used this nanocomposite in the urchin-like structure to develop a label-free device on a GE with targeted nucleic acid via DNA hybridization. Farshchi et al. [120] electrodeposited core–shell Ag and AuNPs onto a graphene quantum dot nanoink. This composite enabled a paper-based electrochemical biosensor for miRNA-21 detection.

It is noticeable that advances in DNA biosensors are directed toward manufacturing devices for label-free detection of target analytes, in which different nanomaterials have been applied to improve the analytical performance of devices without the necessity of labels. Furthermore, DNA biosensors offer a high degree of versatility, once various targets can interact with complementary nucleic acids, enabling biosensors to assist in specific and preventive diagnoses. Additionally, these devices allow for the sensitive and selective monitoring and detection of biomolecules. They are also easy and quick to fabricate, exhibit greater stability against physical and chemical variations, and are relatively cost-effective.

It should be noted that, even beyond the advances in DNA biosensors, the growing use of new materials for their manufacture corroborates a future of potential new devices developed for this field. Although some possible cost limitations and the difficulty in transporting the devices for applications such as self-testing, the variety of materials used in the current sensors mentioned indicates progress in the field and interesting solutions for the final application to become a commercial reality. Some DNA biosensors described in this section are summarized in Table 4.

Regarding genosensors, recent research using GCE is also evident, demonstrating the electrode’s analytical capabilities in terms of sensitivity and robustness. However, it is important to highlight that the area of disposable, portable, and miniaturized sensors shows initial growth that promises long-term advancement. Resistance to disposable sensors, typically printed, stems from their limitations in sensitivity and lack of high reproducibility on a large scale, particularly for carbon-based sensors produced manually in laboratories. While genosensors demonstrate highly satisfactory and reproducible analytical responses, the shift to simple, large-scale production sensors has not yet gained widespread adoption among major research groups worldwide, who often prefer the use of precious metals, which increase system costs. The number of lab-made devices produced, mainly since 1980 [123], corroborates the current stage of devices that seek system miniaturization, portability, data collection via smartphones rather than large benchtop equipment, and, above all, PoC potential. This advancement, which still has sufficient room for expansion and scientific progress, is also indicated by the high financial value generated by label-free electrochemical biosensors and new device structures, increasingly detaching themselves from conventional electrodes. These topics will be discussed below.

## 4. Multiplex Assays by Using Label-Free Electrochemical Biosensors

Corroborating the expansion of the label-free biosensor and the satisfactory results and potential application for DNA/RNA devices and other bioreceptors, new application/determination perspectives have been gaining interesting advances. In this sense, the new approach of label-free electrochemical sensors presents significant challenges and opportunities in the context of multiplex assays [124]. In this context, multiplex assays can be particularly useful for applications such as detecting infectious diseases like Zika and Dengue, diagnosing certain types of cancer, or identifying personalized therapies. Recent studies suggest that, although this technology has shown promise, its application in multiplex assays still faces limitations regarding signal complexity and the ability to simultaneously distinguish multiple analytes with high precision [125].

**Figure 3:**
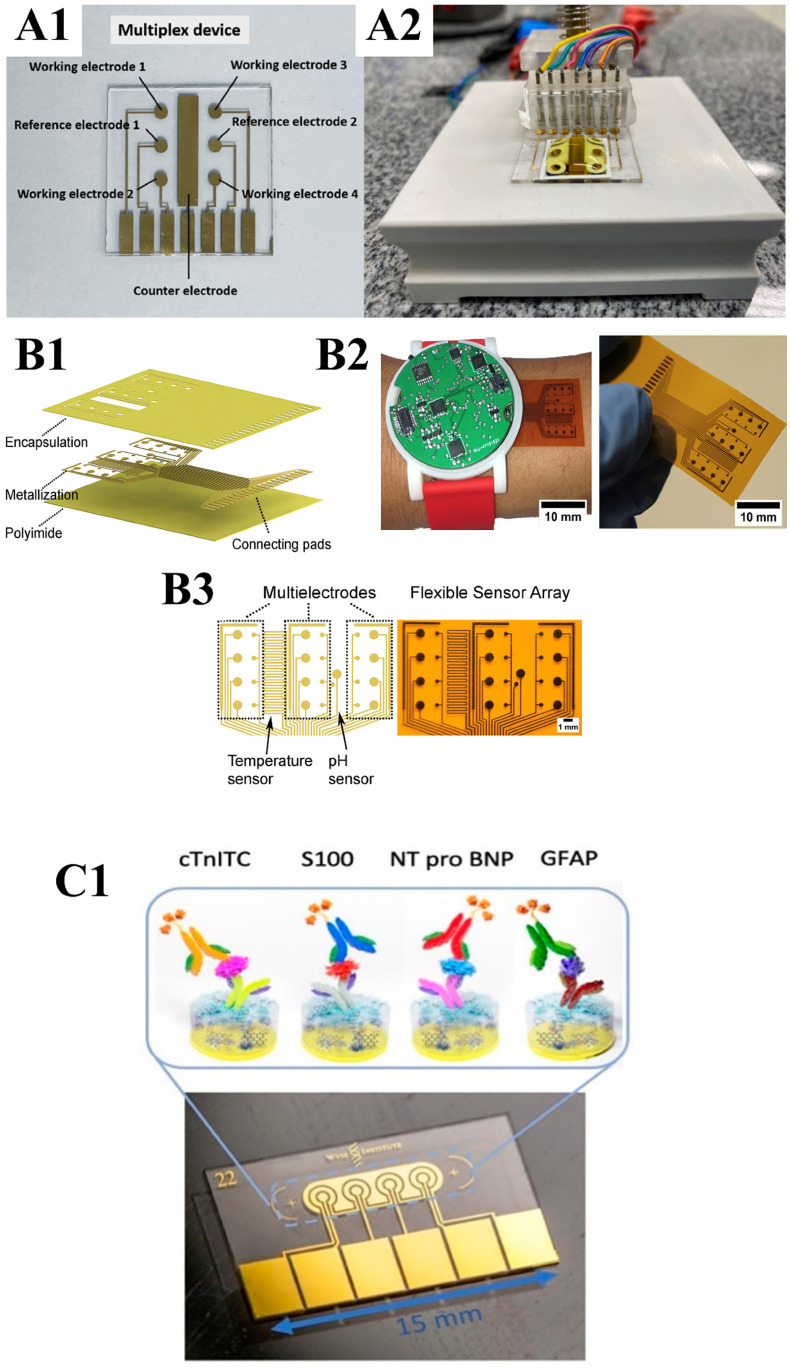
Designs of Label-Free Electrochemical Sensors for Multiplex Assays (**A1**) Gold multiplex device with four WEs, two REs, and one CE; (**A2**) Measurement setup showing the electrolyte solution over the multiplex device and the connector linking the electrodes to the potentiostat; Reproduced with permission from Elsevier ref [126]; (**B1**) Exploded view of the flexible sensor array displaying the distinct layers: polyimide at the base, metallization in the middle, and encapsulation on top; (**B2**) Image of a wrist-worn wearable multiplexing system housed in a 3D-printed watch case for demonstration purposes; Image of the flexible sensor array. (**B3**) CAD model of the flexible sensor array; Modified and reproduced with permission from Elsevier ref [127]; (**C1**) Diagram of 4-channel multiplexed detection on a single chip. Modified and reproduced with permission from ref [128].

The electrochemical biosensors described in recent studies, like those by Sampaio et al. [126], demonstrate significant advances in detecting multiple biomarkers on a single platform. These biosensors utilize the NS1 while achieving high specificity and sensitivity. The biosensor device features four WEs, two reference electrodes (RE), and one counter-electrode (CE). To ensure precise electrode spacing, RE 1 (on the left) monitors WE 1 and 2, while RE 2 is used for WE 3 and 4. The multiplex was created through photolithography and metal sputtering (Figure 3(A1,A2)).

The unique design of these multiplex devices allows for simultaneous detection of up to four diseases using independent electrodes, making them ideal for PoC diagnostics. These biosensors ensure efficient surface modification and reliable biomarker detection by utilizing SAMs on GE. The portability, cost-effectiveness, and ease of use make this technology particularly promising for rapid diagnostics in regions where these diseases co-circulate. Moreover, the flexibility of the platform allows for the addition of other disease-specific biosensors, further enhancing its potential for real-time health monitoring in resource-limited settings. In addition, it offers a portable PoC diagnostic tool, potentially extendable to detect other diseases with overlapping symptoms.

Yokus et al. [127] developed a wearable, flexible, non-invasive, multiplexed label-free electrochemical biosensor array for continuous sweat monitoring of glucose, lactate, pH, and temperature. The system uses redundant (WEs to improve reliability by averaging outputs, and each channel operates as a three-electrode cell (four WEs, one RE, and one CE) connected to a potentiostat via a multiplexer (Figure 3(B1–B3)). Its analytical performance was comparable to running tests separately and was validated for simultaneous measurements with high sensitivity and specificity.

In this context, developing multiplex assays for electrochemical sensors, such as wearable systems, improves the simultaneous detection of multiple analytes. This approach offers significant advantages for PoC diagnostics and real-time health monitoring, especially in areas with limited medical resources, where rapid multi-target detection is crucial.

This new perspective aims to simplify diagnostic tools by leveraging label-free, electrochemical detection methods, making them more accessible and scalable. Indeed, hybrid multiplexing topology can enhance real-time performance while minimizing circuit complexity, paving new opportunities in electrochemical sensor applications within wearable health technologies.

Numerous techniques have been developed for the modification of electrode surfaces. However, their effectiveness is often undermined by problems such as reduced sensor conductivity, complex manufacturing processes, and poor antifouling properties. These limitations, combined with persistent problems such as biofouling and the resulting reduction in sensor sensitivity, have significantly hindered the widespread use of electrochemical sensors in clinical diagnostics. Timilsina et al. [128] showed that antifouling nanocomposite coatings can enable multiplexed electrochemical sensors to maintain high sensitivity and specificity in complex fluids such as blood and plasma (Figure 3(C1)). They developed a 3D coating based on BSA interlaced with conductive nanomaterials and functionalized with bioreceptors. This architecture improves electron transfer while reducing nonspecific binding and fouling.

This has enabled the development of multiplex platforms that integrate these sensors with microfluidic systems to simultaneously detect multiple clinical biomarkers, such as those for sepsis and myocardial infarction, with impressive results regarding LOD and specificity. Such systems offer a cost-effective, scalable solution for PoC diagnostics, capable of being expanded to detect multiple biomarkers on a single platform, representing a significant advance for personalized medicine.

In this way, label-free electrochemical sensors represent a significant innovation in multiplex assays, offering the potential for highly sensitive, selective, and scalable diagnostic platforms. As advances in antifouling techniques and sensor design continue, these technologies will play a crucial role in personalized medicine and PoC diagnostics, particularly in resource-limited settings. The ability to detect multiple biomarkers on a single platform, with the portability and flexibility to adapt to different diseases, positions this approach as a key driver in the future of healthcare diagnostics.

However, although multiplex systems for electrochemical biosensors theoretically present advantages, such as multiple electrodes in a single system capable of detecting different biomarkers in a single analysis, practical implementation often presents obstacles to the robust advancement of this methodology. When adding more than one WE, it is necessary to consider interferences within the system itself, particularly when only one CE and one RE are used for multiple WEs, resulting in measurements with slightly different analytical responses due to the complexity of the operation. One approach is the production of a sensing platform with multiple electrode systems integrated into the platform, but with independent analyses. However, in this scenario, the question arises as to whether it is really necessary to add multiple systems to a platform rather than performing different analyses in a single three-electrode system, for example. Finally, although growth in multiplex systems is evident and this tool emerges as a way to diagnose a wide range of diseases in a single system, the existing problems indicate that more in-depth studies are necessary. In this sense, the works cited here address the limitations and seek to overcome them, which guarantees scientific advancement. The limitations pointed out are not a factor that eliminates the use of multiplex systems, since there are studies that show their efficiency, but rather raise the debate on how to overcome these limitations in order to make them more broadly applicable. Thus, a broad area opens up that can utilize new integrated equipment, artificial intelligence for multifactorial analyses and advanced data processing. a key driver in the future of healthcare diagnostics.

## 5. New Applications of Label-Free Devices for Medical Diagnosis

As cited in previous sections, recent advances in label-free electrochemical devices have shown potential for medical diagnostics. One of the most prominent applications is the detection of viral infections such as dengue, influenza, Ebola, HIV/AIDS, and COVID-19 [129]. Due to the rapid response of on-site virus detection, with precision and low cost, electrochemical biosensors have gained increasing importance.

In 2023, dengue recorded more than 6.5 million cases and 7300 deaths in more than 80 countries [130]. An innovative DNA spin hybridization-based biosensor has been developed to detect specific DNA sequences using properties of spin-polarized electrons for more sensitive and selective detection [131]. A label-free genosensor was developed to detect Dengue virus RNA quickly without nucleic acid amplification, using SAMs of 6-mercaptohexanoic acid (MHA) and 6-mercapto-1-hexanol (MCH) to optimize probe DNA density on GE [132]. The method showed high sensitivity and could distinguish between DENV serotypes. An electrochemical immunosensor was developed to detect NS1 glycoprotein in serum and urine, using a GE modified with anti-NS1 antibodies and a poly-ethylenediamine (poly-EDA) film [133]. For the cases mentioned, there are different platforms and materials used that contribute to a range of applications for clinical diagnostics, whether it be the use of new materials or different surfaces for the sensors.

**Figure 4:**
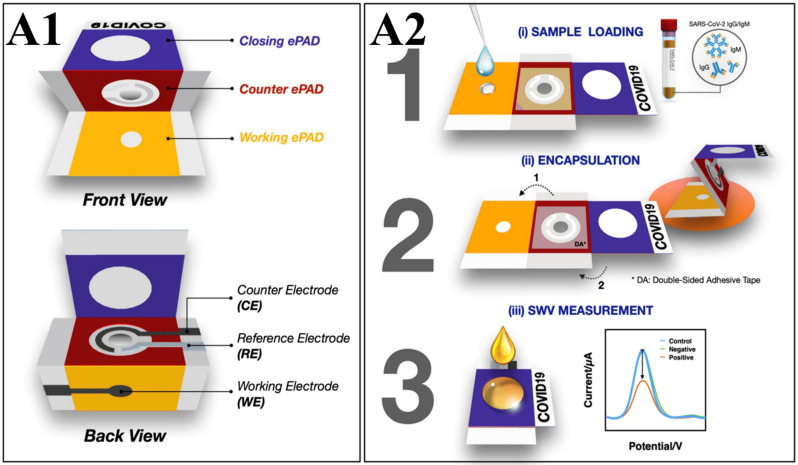
Schematic illustration of the (**A1**) device components; (**A2**) detection principle. Reproduced with permission from Elsevier ref. [136].

COVID-19, caused by the SARS-CoV-2 virus, has become a global threat, infecting 770 million people and causing over 7 million deaths [134,135]. The pandemic created an urgent need for devices that could quickly, accurately, sensitively, and simply determine if an individual was infected, enabling timely emergency medical measures. An electrochemical biosensor built on paper was developed to rapidly detect IgG against the S1 protein within 30 min, aiding in the timely diagnosis and management of COVID-19. The electrochemical paper-based device (ePAD) (Figure 4(A1)) consists of three foldable layers: a WE, a CE, and a RE. Using paper as the substrate makes the device low-cost, portable, and safely disposable by incineration [136]. SARS-CoV-2-IgM and SARS-CoV-2-IgG antibodies were immobilized on the ePAD test zone (Figure 4(A2)) to detect the virus. Antibodies binding to the S1 protein showed that IgM (~900 kDa) had better sensitivity than IgG (~150 kDa), likely due to its larger size [137,138,139]. The antibodies were immobilized on a hydrophilic paper zone using a rGO-EDC/NHS layer [140]. The device was folded to reduce contact with biohazard fluids.

A new faradaic impedimetric immunosensor was tested for clinical analysis of COVID-19 antibodies. This biosensor uses SAM-modified mercaptohexanoic acid interdigitated electrode arrays (IDA) to improve sensitivity and reliability in detecting S1 protein antibodies [141]. The microarray structure significantly affects the sensor’s electrochemical behavior, and the study addresses baseline signal drift issues in redox systems, likely due to electrode corrosion [142].

Another infection that has gained prominence in the last 20 years is *Escherichia coli* (*E. coli*) O157:H7. The contamination of water sources is a global problem that affects both developed and developing countries. Ingestion of food and water contaminated with *E. coli* O157:H7 seriously threatens public health and causes significant financial losses. This pathogen is one of the most common and lethal enterohemorrhagic pathogens, particularly affecting infants, children, immunocompromised individuals, and the elderly. Shiga toxins are those produced by these bacteria that destroy cells in the intestinal mucosa and lead to fatal health conditions. Electrochemical biosensors are ideal for detecting pathogens in contaminated water and food due to their miniaturization, flexibility, and ease of use. Various devices have been developed to detect *E. coli* O157:H7 in different samples using methods like DPV, EIS, CV, and SWV [143,144,145]. A modified GCE with a nanocomposite coating of rGO, polyvinyl alcohol, and AuNPs was used to detect *E. coli* O157:H7 in tap water, milk, and meat samples [146].

In the context of virus diagnostics, real-world PoC applications are more commonly encountered in everyday life, such as rapid tests for COVID-19 and influenza. Since this review prioritizes electrochemical analyses, it is important to highlight that the sensors commonly found in pharmacies or clinical settings are predominantly colorimetric and usually qualitative. There are still limitations to the use of electrochemical sensors in routine practice, with the glucometer being one of the best-known examples that meets this demand. Therefore, we aim to highlight new biosensors for viral diseases, which are currently limited to benchtop testing, with little propensity for real-world application and for obtaining quantitative results. Unfortunately, this remains a recurring limitation in discussions of impedimetric or amperometric electrochemical biosensors, although it has been advancing over time. Thus, although advances in materials science for the production of these biosensors are remarkable, their applicability remains confined to the laboratory bench.

Research using PoC technology to diagnose biomarkers of neurodegenerative diseases has also gained prominence in recent years. This is especially relevant for neurodegenerative diseases such as Alzheimer’s and Parkinson’s, where early detection of biomarkers can be crucial for the treatment and management of the disease. PoC analytical tools facilitate access to diagnosis and significantly improve patients’ quality of life by enabling faster and more personalized interventions.

Alzheimer’s disease is the seventh leading cause of death worldwide and has seen a significant rise in prevalence over the past 25 years. A potential treatment currently under investigation involves targeting regulatory genes, such as miRNAs, to address the disease [144,147]. A device was developed to capture miRNA-206 using MCH and 5′thiolated miRNA [148,149]. The study successfully optimized the methodology for ultrasensitive target detection, identifying miRNA-206 in real plasma samples from AD patients, with concentrations in the nmol L^−1^ range.

Parkinson’s and Alzheimer’s diseases have α-Synuclein as a biomarker [150,151]. A study developed a label-free immunosensor using electrolyte-gated organic field-effect transistors for detecting this protein [143]. Parkinson’s disease is characterized by movement issues, mental health problems, sleep disturbances, and an electrochemical bioassay for early detection targeted α-synuclein using a modified GCE [147,152].

Hereditary Huntington’s disease is caused by a mutation in the HTT gene, leading to excessive CAG trinucleotide repeats and nerve cell degeneration in the brain [143]. A device was developed to detect these repeats using a peptide nucleic acid (PNA) probe (CTG-6) immobilized on a gold layer. Another study focused on reducing secondary gene structures and false positives in detecting the CGA repeat sequence [145].

Label-free diagnostics for heart diseases are advancing, with electrochemical sensors focusing on cardiac troponin 1 [153]. An electrochemiluminescent (ECL) immunosensor using CdS-MoS_2_ composites showed high sensitivity and accuracy in detecting troponin 1 in human serum samples. Another system, a label-free photoelectrochemical (PEC) device, used CdS nanowires and SnNB206 nanosheets to simultaneously detect cTnI and myoglobin (Myo), demonstrating good reproducibility and accuracy in real samples [154]. The devices were tested on real samples, showing good reproducibility and accuracy.

While it is possible to find truly interesting work on the detection of biomarkers for neurodegenerative or cardiac diseases, there is still a limitation related to the predominant use of conventional electrodes, even when nanomaterials such as gold, MWCNTs and graphene are employed. A real constraint is the lack of studies that gain prominence using materials such as metal oxides, other carbon allotropes and MOFs, for example. Thus, even though it is possible to affirm the exponential advance in the search for more sensitive, cheaper and more portable label-free electrochemical biosensors, there remains a restriction to a narrow set of commonly used materials and limited opportunity for the exploration of less conventional ones. However, it should be noted that the results of the biosensors produced and highlighted in this work contribute to advances in the area of label-free diagnostics, but do not address or discuss their potential for real-world application, rapid diagnosis and use in places of need.

Table 5 summarizes the main characteristics and analytical parameters for the label-free devices cited here. The selected studies have linear range and LOD values comparable to other reports in the literature that aim to determine the same biomarkers. However, one of the columns in the table compares the selected studies according to whether the authors address the potential of the developed biosensors for PoC applications. For studies with a negative answer, this is not a judgment on how the authors interpreted their own data, but merely a highlight that, in these cases, the discussion was limited to the development of a new label-free biosensor that showed improved analytical response, either in sensitivity or in lower LOD. Similarly, when the answer to the question is “yes,” this does not necessarily mean that the authors discussed the new device in terms of its real-world PoC application, but rather that, after demonstrating a lower linear range or LOD, they indicated the potential for system portability and future studies aimed at PoC use.

This comparison shows that, whether or not the authors mention the potential of the biosensors for PoC, the discussion usually ends with a possible future application and does not delve into the commercial potential of the device. Therefore, there is a lack of studies that propose new devices and effectively discuss their fabrication, testing and application in real conditions with a focus on commercialization. The discussion is almost always limited to statements such as “the sensor demonstrated potential for future applications in clinical diagnostics,” even when the analytical results are superior to those of earlier studies already reported in the literature.

Recent advances in PoC technologies in conjunction with label-free devices have significantly improved the ability to respond quickly and accurately to critical public health situations, especially for emerging diseases. These diagnostic devices have shown clinical efficacy and the potential to transform disease management in various settings, from urban clinics to remote areas with limited resources. The growing demand for rapid and accessible diagnostics, along with technological advancements, presents new opportunities for commercialization and large-scale adoption. The following discussion will cover market trends, challenges, and opportunities shaping the future of PoC devices, emphasizing their potential to revolutionize healthcare and provide innovative solutions for diagnosing emerging diseases.

## 6. Commercial Potential for Label-Free Devices

The development of modern biosensors shows tremendous potential for clinical diagnostics. The biosensor market is projected to surpass $28 billion by 2024, with a forecasted growth rate of 15 to 20% per year [155]. The emerging field of flexible and disposable wearable sensors, which combines microfluidics and electronics, holds promise for the future of non-invasive wearable diagnostics. Biosensors are generally very affordable, often costing less than 1 USD per device, as they require micro volumes of samples and reagents for performing the test [156]. Zhang and Wang [161] highlighted in their work the global market trend regarding the application of label-free immunosensors, demonstrating a 9% growth in recent years, in addition to an expected economic turnover of almost US$3 billion by 2025. In this sense, it is important to highlight the significant increase in investment in the development of label-free biosensors and the related financial activity in this field. This trend is closely linked to the search for devices capable of enabling early and rapid clinical diagnosis; however, in the studies surveyed in this review, most of these platforms remain at the PoC stage, with their application still largely confined to research laboratories.

Label-free electrochemical biosensors are highly suitable for PoC diagnostics, as they do not require qualified technicians and can be used by patients even in the early stages of disease. Despite extensive research, few label-free electrochemical biosensors are commercially available due to obstacles outside academia. Key issues include the stability of the biorecognition layer, which impacts the device’s lifespan, and the high cost of complex or expensive materials [162]. Regulatory approval processes also add to the final product’s cost. Improved collaboration between universities and industry is needed for better prototype testing and validation. Also, high-performance devices face challenges in uncontrolled environments, such as interfering species, biofouling during sampling, or complex formations [157]. Considering that commercially applied diagnostic devices are mostly colorimetric, often using lateral-flow or microfluidic approaches, the difficulty for electrochemical sensors in being applied as wearable devices or in PoC systems lies in the way the electrochemical signal is transduced and converted into a simple, user-friendly readout. In many cases, the generated data ultimately need to be reduced to a response similar to that of the most well-known biosensor worldwide, the glucometer, that is, a single quantitative value derived from an underlying electrochemical measurement.

As an alternative to these mentioned challenges, low-cost options for electrode production, such as screen or 3D printing, positively impact the reduction in production costs, thereby increasing their accessibility [163]. With a view to large-scale production, screen-printing, a method developed to assist the expansion of the market for smart wearable devices that require lightweight, high-performance, and outstanding mechanical flexibility, is widely used to create low-cost analytical platforms [164].

Other useful tools are the microfluidic or lateral flow devices. However, in this review, although we recognize the great importance of such devices for clinical diagnosis, we do not focus on increasing their representation, as we consider that direct comparison with electrochemical sensors falls outside the scope of our discussion. Flow-based and lateral-flow systems inherently have facilitated portability because they do not require on-board electronic devices for data interpretation, which makes their technological challenges and development pathways substantially different from those of electrochemical platforms. These platforms can be constructed with simpler equipment, such as double-sided pressure-sensitive adhesives and polystyrene sheets [158]. These microfluidic examples are assembled by gluing laser-cut double-sided tapes to polystyrene caps, with sample entrances made from cut pipette tips attached with epoxy adhesive. The flow system is sealed over the electrode system to integrate the microchannels properly. Also, metal electrodes were created using hollow adhesive masks glued to thoroughly clean and laser-cut glass coverslips. This method ensured precise and reproducible electrode designs for the C_4_D system’s performance.

It is also relevant to mention that in electrochemical biosensors, a process known as biofouling can occur, which occurs when macromolecules of complex biological fluids, such as blood, saliva, and urine, adhere to the surface of the sensor, forming a layer that interferes with its function [165]. This phenomenon reduces the sensitivity, reproducibility, and reliability of sensors, making it difficult to detect analytes accurately. To combat biofouling, researchers are developing anti-biofouling strategies, such as modifying sensor surfaces with antifouling materials, to improve the performance and reliability of devices in complex biological environments.

Another tool that can help in the process of making large-scale production of label-free PoCs economically viable is artificial intelligence (AI), which can optimize manufacturing processes to reduce costs. The advent of artificial intelligence has become relevant to automating these process improvements. Still, this integration has not yet been fully realized and is seen as a gap to be filled. AI can also significantly improve data analysis in biosensor applications, aiding in automatic signal processing, interpreting large amounts of real-time monitoring data, and enabling the prediction of biological events [124,138]. However, there is a gap in the effective implementation of AI algorithms that can process complex biosensor data in real-time.

While some PoC show better sensitivity than conventional methods, they often lack the robustness for mass use. Incorporating nanomaterials has improved their analytical performance, but many devices have only been tested on model samples, not real-world applications [137,138]. Real-world testing is crucial for validating their effectiveness and reliability [99,139]. So, improving manufacturing processes to ensure reproducibility is essential for commercial viability.

In label-free devices, the stability of the bioreceptors is crucial to ensure the accuracy and reliability of measurements. Many commonly used bioreceptors can degrade or lose their biological activity over time, especially under harsh environmental conditions [166]. This results in a lack of stability that compromises the sensitivity and reproducibility of the sensors. However, DNA stands out as a highly stable bioreceptor. Due to its robust structure and ability to maintain integrity in a wide range of conditions, DNA is less susceptible to degradation. This high stability makes DNA an ideal choice for label-free devices, allowing for more accurate and consistent detections over time.

However, even when highly stable bioreceptors such as DNA are employed, several system-level factors still limit the translation of label-free electrochemical biosensors beyond the research laboratory. The transport of these devices, their proper storage, and the development of integrated kits that minimize or eliminate sample pre-treatment before sensor application, together with compact and reliable electronic hardware for signal transduction and data processing, remain critical bottlenecks for large-scale commercialization. Although the absence of labeling enables more direct and rapid measurements at the sensing interface, the surrounding requirements in terms of logistics, sample handling and instrumentation continue to slow the transition of these platforms to real-world applications.

The market for multiplex devices is experiencing significant growth due to their ability to reduce diagnosis time and costs by performing multiple analyses on a single platform. These devices provide a comprehensive view of patient health, use minimal sample volumes, and have high sensitivity for detecting diseases at the pg mL^−1^ level [167,168]. Challenges remain, such as interactions with complex biochemical environments and target markers. Key features like safety, cost-effectiveness, miniaturization, in situ measurement, and short analysis times highlight their potential in the future of medical diagnostics. From these observations, it can be emphasized that label-free devices already present sufficient analytical performance for commercial use. Yet some obstacles persist, including the need for more robust methods, affordable production costs, and scalable mass production. Despite their enormous commercial potential, these devices still strive for more space in the industry and have much to contribute to society. These devices must move from academia to products through entrepreneurship, the creation of start-ups, and partnerships with companies already known in the sector [134].

## 7. Conclusions and Perspectives

Although their first application dates back to the 1980s, label-free devices have seen new advances and reinventions over the years. With this in mind, this review aims to highlight studies emphasizing the importance of developing label-free devices and looking for new architectures that make their commercial and PoC applications feasible. Given the relevance of their advantages, such as simplicity, relatively low cost, high sensitivity, potential for post-analysis disposal, low consumption of solution and reagents, miniaturization and portability of the system, and rapid response, label-free electrochemical sensors stand out for their wide range of applications, mainly of a clinical nature.

Thus, this review has compiled different works focused on label-free electrochemical sensors, covering different interesting works, including genosensors based on DNA and other receptors, as well as addressing other potential aspects in the development of label-free devices by introducing their use in multiplexed systems and their commercial potential. Overall, this work highlights the importance of directly determining biomarkers concomitant with the emerging need for affordable devices. To complement the advantages and the scientific progress achieved in this field, we emphasize a key limitation: in most of the studies surveyed in this review, the work is conceived and completed within the research environment without a deeper discussion of the real-world, and, in particular, commercial application potential of new label-free electrochemical biosensors. Analytical performance is not in question here, since these studies consistently present new methodologies, novel materials and components, and demonstrate high sensitivity, selectivity for different biomarkers, and good reproducibility. Rather, we highlight the difficulties associated with the system-level requirements surrounding the application of these devices and their commercialization.

As a perspective for this area, our analysis indicates several priority directions for the development of label-free electrochemical sensors:

- The first is the need to reconcile large-scale production, bioreceptor stability and the logistics of transport and storage in a way that guarantees device quality and applicability for end users. For most emerging platforms, the storage of new electrochemical devices and their reliable performance in real samples remain stages that require substantial progress.
- A second key point is the integration of technologies that allow for PoC testing without relying on specialized benchtop instrumentation, with real potential for the use of smartphones and artificial intelligence-based software for signal processing and data analysis. In recently published works included in this review, the analytical capabilities of label-free biosensors are demonstrated; however, the integrated PoC system is not fully addressed. Nevertheless, it is important to highlight that devices already exist that offer a microstation or the potential for smartphone use, contributing to the advancement of this area of PoC, but data interpretation and manipulation by non-specialized personnel require further development [169,170,171,172,173].
- Third, increased robustness and accessibility are essential to ensure effective application in clinical analyses. This includes not only intrinsic analytical robustness, but also validation in real matrices, interlaboratory comparability and cost structures compatible with routine diagnostics. In this sense, some recent studies propose promising alternatives, but they are still far from representing the dominant trend in the field.
- Finally, expanding device architectures for simultaneous detection of multiple markers in multiplex systems remains a critical frontier. Such systems could support PoC diagnosis for different diseases or multiple biomarkers of a single disease within one platform. We have observed initial growth in this area, but substantial room remains for innovation in electrode design, signal deconvolution and data handling.

Although these challenges still need to be addressed and improved in future work, this review has sought to compile and discuss studies that contribute to the advancement of label-free electrochemical biosensors and to articulate our perspective on their relevance and potential application across a wide range of emerging diseases and other areas of research. In particular, we emphasize that further progress will depend less on incremental gains in analytical figures of merit and more on overcoming system-level barriers to real-world and commercial implementation.

## Figures and Tables

**Table 2:** Recent studies on other bioreceptor-based label-free detection sensors.

Bioreceptors	Targets	Detection Range	LOD	Ref.
Antibodies	plasma-based Aβ1–42	11 pmol L^−1^ to 55 nmol L^−1^	2.398 pmol L^−1^	[49]
	S1 protein	5.0 to 75.0 nmol L^−1^	1.36 nmol L^−1^	[47]
	S1 protein	1.0 to 75.0 nmol L^−1^	0.30 µmol L^−1^	[46]
	Tiamulin (TML)	0.01 to 1000 ng mL^−1^	0.003 ng mL^−1^	[48]
	A29 protein	1.8 to 100 ng mL^−1^	0.48 ng mL^−1^	[54]
	human epidermal growth factor receptor 2	0.1 to 100 nmol L^−1^	0.23 fmol L^−1^	[55]
	Oncostatin M	37 to 1000 pg mL^−1^	2.86 pg mL^−1^	[56]
Aptamers	Microcystin-LR (MC-LR), Cylindrospermopsin (CYL), anatoxin-α, saxitoxin, and okadaic acid (OA).	0.073–150 nmol L^−1^, 0.018 to 200 nmol L^−1^, 0.018 to 200 nmol L^−1^, 0.018 to 200 nmol L^−1^, 0.018 to 200 nmol L^−1^, respectively	0.0033, 0.0045, 0.0034, 0.0053, and 0.0048 nmol L^−1^, respectively	[43]
	Atrazine	1.0 to 250 nmol L^−1^	0.06 nmol L^−1^	[44]
	hemoglobin A	13.5 to 150 nmol L^−1^	0.85 pg mL^−1^	[57]
	S. aureus	10 to 10^8^ CFU mL^−1^	3.0 CFU mL^−1^	[58]
	Tau381	1.0 to 100 pmol L^−1^	0.7 pmol L^−1^	[59]
	Oxytetracycline	1.0 to 540 nmol L^−1^	30 pmol L^−1^	[60]
MIPs	α-amylase	6.0 × 10^−6^ to 0.60 mg mL^−1^	<3.0 × 10^−4^ mg mL^−1^	[51]
	Homocysteine	5.0 to 150 µmol L^−1^	1.2 µmol L^−1^	[61]
	L-tyrosine	100 pmol L^−1^ to 5 mmol L^−1^	10 pmol L^−1^	[62]
	Dopamine	0.005 to 100 μmol L^−1^	0.0006 μmol L^−1^	[63]
	Glucose	0.001 to 10 mmol L^−1^	0.26 nmol L^−1^	[64]
	Testosterone	1.0 to 25 ng dL^−1^	1.0 ng dL^−1^	[65]
	cortisol	0.005 to 5000 ng mL^−1^	0.019 pmol L^−1^	[66]

**Table 3:** Brief survey of different transducers for immunosensors and their applications for label-free determination of different biomarkers.

Type of Device	Immunosensor	Target	Technique	Linear Range	LOD	Samples	Possible Portability?	Ref.
GCE	AuNPs/CCNTs/CS nanocomposite	carcinoembryonic antigen	CV and SWV	0.001–400 ng mL^−1^	~1 pg mL^−1^	Real human serum	No	[85]
	AuNPs/pDa/rGO	NS1	DPV	0.001–100 μg mL^−1^	0.001 μg mL^−1^	not tested	No	[86]
	COFe_2_O_4_-C60NP	Anti-A2	DPV	0.1 fg mL^−1^–0.1 μg mL^−1^	0.1 fg mL^−1^	sheep blood serum	No	[87]
SPE	ESPEC	A-synuclein	SWV and EIS	0.01–100 ng mL^−1^	4.1 pg mL^−1^	Fetal bovine serum	Yes	[88]
	PEI/ABsa/PSS	S. aureus	EIS	10^4^–10^7^ CFU mL^−1^	1000 CFU mL^−1^	Spiked PBS	Yes, and they tested the portability.	[89]
	LIG immunosensor	DENV	DPV	25–2000 ng mL^−1^	17.40 ng mL^−1^	Real human serum	yes	[90]
GE	Sulfo-LC-SPDP	SPB	EIS and CV	2–2000 ng mL^−1^	0.1 ng mL^−1^	Real Amniotic Fluid	yes	[91]
	COOH-CNF/Ti_3_C_2_T_x_MXene/PANI-AgNPs	OPG	DPV	0.01–1000 fg mL^−1^	0.00194 fg mL^−1^	Real human serum	No	[92]
3D	PLA-G	PARK7/DJ-1	EIS and CV	5.0–200 μg mL^−1^	1.01 μg mL^−1^	Commercial Human serum and synthetic cerebrospinal fluid	No	[93]
	CB-PLA	S1 protein	CV	0.01–4.5 nmol mL^−1^	2.7 pmol mL^−1^	Commercial human serum and synthetic saliva	Yes	[94]
	CB-PLA Protopasta	TNFα	DPV	160–1820 pg mL^−1^	44.5 pg mL^−1^	Real faecal pellets	No	[95]

AuNPs—gold nanoparticles; CCNT—coiled carbon nanotubes; CS—chitosan; pDa—polydopamine; rGO—reduced graphene oxide; COFe_2_O_4_-C60NP—cobalt-hydrocarbon iron oxide nanocomposite; ESPEC—screen-printed carbon electrode modified with carbon nanotubes and electrodeposition of AuNPs; PEI—polyethyleneimine; PSS—poly(4-sodium styrenesulfonate); LIG immunosensor—CO_2_ laser immunosensor; Sulfo-LC-SPDP—(sulfosuccinimidyl 6-(3′-(2-pyridyldithio)propionamide)hexanoate); COOH-CNF—carbon nanofibers; Ti_3_C_2_T_x_MXene—two-dimensional transition metal carbides/nitrides (MXenes) using titanium; PANI-AgNPs—polyaniline nanoparticles; PLA—polylactic acid; CB—carbon black; CB-PLA Protopasta—commercial filaments.

**Table 4:** Genosensors manufactured with different modifiers for signal mediation and amplification.

Types of Mediators and Amplifiers	Modifiers	Targets	Detection Range	LOD	Ref.
Genosensor	DNA capture sequence and 2-mercaptoethanol	SCA sequence gene	0.1–7.5 μmol L^−1^	7.0 nmol L^−1^	[102]
Redox mediators	Methylene blue	MicroRNA let-7a	0.001–1000 pmol L^−1^	0.45 fmol L^−1^	[114]
	Ferrocene	cTnI	10 fg mL^−1^–10 ng mL^−1^	2.6 fg mL^−1^	[115]
Mimetic enzymes	Tripedal DNA walker (MB1, MB2, MB3)	Tau protein	0.1 fmol L^−1^–1.0 nmol L^−1^	0.43 fmol L^−1^	[116]
	Tripedal DNA walker (HP1, HP2,HP3)	α-synuclein oligomer	1.0 fmol L^−1^–10 pmol L^−1^	0.46 fmol L^−1^	[118]
Nanomaterials	Gold nanocubes	Prostate cancer gene short sequence and EPI anti-cancer drug	0.04–0.8 μmol L^−1^ 0.8–20 μmol L^−1^	0.01 μmol L^−1^	[110]
	Zinc oxide and AuNPs	*Mycobacterium tuberculosis*-DNA	2.5–250 pmol L^−1^	1.8 pmol L^−1^	[119]
Carbon nanomaterials	Single-wall carbon nanotubes	Doxorucibin	1.0 nmol L^−1^–20 μmol L^−1^	<0.6 nmol L^−1^	[121]
	Nanocomposite of graphene and MWCNTs	Oligonucleotide/NHL gene	1.0 fmol L^−1^–1.0 nmol L^−1^	0.5 fmol L^−1^	[122]
Hybrid nanocomposites	Nanoclusters of AuNPs and CNTs	DNA hybridization	0.1 pmol L^−1^–10 nmol L^−1^	5.2 fmol L^−1^	[117]
	Core–shell silver and AuNPs electrodeposited on a graphene quantum dot nanoink	miRNA-21	5.0 pmol L^−1^–5.0 μmol L^−1^	-	[120]

**Table 5:** Label-free devices applied to the determination of several disease biomarkers.

Diseases	Target	LOD	Linear Range	Discussing the Potential for PoC?	Ref.
Dengue	Virus genomic sequence	0.12 pmol L^−1^	-	No	[131]
Dengue and Zika	NS1	6.8 ng mL^−1^	20–800 ng mL^−1^	Yes	[133]
Dengue and Zika	NS1ZV NS1DV	0.54ng mL^−1^(Zika) 1.17 ng mL^−1^(Dengue)	15.62–1000.00 ng mL^−1^ 15.62–500.00 ng mL^−1^	Yes	[126]
Dengue	DENV1 RNA	20 PFU mL^−1^	102 to 105 PFU mL^−1^	No	[132]
COVID-19	IgG	0.2 ng mL^−1^	1.37–145 ng mL^−1^	Yes	[155]
COVID-19	IgG IgM S1 protein	0.96 ng mL^−1^ 0.14 ng mL^−1^ 0.11 ng mL^−1^	- - 1–1000 ng mL^−1^	Yes	[136]
COVID-19	SARS-CoV-2 Antibody	21 ng mL^−1^	50–105 ng mL^−1^	Yes	[141]
*E. coli*	O157:H7	9.34 CFU mL^−1^	9.2–9.2 × 10^8^ CFU mL^−1^	No	[146]
*E. coli*	O157:H7	2 CFU mL^−1^	10–1000 CFU mL^−1^	No	[156]
Enterovirus 71	EV71	0.1 ng mL^−1^	0.1–6000 ng mL^−1^	Yes	[157]
Huntington	Repeated sequences of CAG	1 pmol L^−1^	1–100 pM	No	[143]
Huntington	Repeated sequences of CAG	100 amol L^−1^	100 amol L^−1^–100 amol L^−1^	Yes	[152]
Alzheimer and Parkinson	α-synuclein	0.25 pmol L^−1^	0.25 pmol L^−1^–250 nmol L^−1^	Yes	[158]
Alzheimer	MiRNA-206	0.15 amol L^−1^	1 µm to 1 amol L^−1^	Yes	[149]
Parkinson	α-synuclein	0.02 ng mL^−1^	0.02–64 ng mL^−1^	Yes	[152]
Acute myocardial infarction	cTnI	1.0 pg mL^−1^	5.0 pg mL^−1^–100.0 ng mL^−1^	No	[159]
Acute myocardial infarction	cTnI	20 fg mL^−1^–2 ng mL^−1^	1.07 fg mL^−1^	No	[160]

NS1, NS1ZV, NS1DV—Dengue and Zika virus antigen; IgG—immunoglobulin G; IgM—immunoglobulin M; S1 protein—SARS-CoV-2 Spike Protein; CAG—trinucleotide; cTnI—cardiac troponin I.

## Data Availability

No new data were created or analyzed in this study.
